# Safety and efficacy of left atrial appendage occlusion with the ACP or Watchman device guided by intracardiac echocardiography from the left atrium

**DOI:** 10.1002/clc.23696

**Published:** 2021-09-08

**Authors:** Thibaut Pommier, Charles Guenancia, Carole Richard, Audrey Sagnard, Marie Fichot, Clemence Salignon‐Vernay, Guillaume Porot, Gabriel Laurent, Luc Lorgis

**Affiliations:** ^1^ Department of Cardiology Dijon University Hospital Dijon France; ^2^ Laboratory of Cerebro‐Vascular Pathophysiology and epidemiology University of Burgundy Dijon France

**Keywords:** ICE, left appendage, local anesthesia, percutaneous occlusion

## Abstract

**Background:**

There is a paucity of randomized data regarding the safety and efficacy of the use of intracardiac echocardiography (ICE) from the left atrium (LA) to guide left atrial appendage occlusion (LAAO) procedures under local anesthesia using either of the available devices.

**Hypothesis:**

The aim of this study was to compare the efficacy and safety of ICE from the LA with transesophageal echocardiography (TEE) for guidance during transcatheter LAAO procedures.

**Methods:**

Single‐center, cohort study of patients undergoing LAAO with the Amplatzer Cardiac Plug or Watchman device. Procedures were guided by ICE from the LA with local anesthesia (*n* = 175) or TEE under general anesthesia (*n* = 49). Efficacy outcomes were procedural success and peri‐device leaks 6 weeks after LAAO. The safety outcome was a composite of procedure‐related complications.

**Results:**

Procedural success was similar between groups: 100% in the TEE‐guided group, and 98% in the ICE‐guided group. Procedure‐related complications such as death, embolism, migration, or major vascular complications occurred similarly between groups (*p* = 0.590). The rate and degree of peri‐device leaks or presence of a thrombus on the device did not differ between groups on follow‐up CT. Turnover time in the catheter laboratory and use of contrast agent were reduced with ICE.

**Conclusions:**

ICE in the left atrium to guide LAAO procedures appears to be as effective and safe as TEE. There was no increase in procedure‐related complications, whatever the device used. ICE resulted in similar procedural success while decreasing procedure time and requiring only local anesthesia.

## 1 INTRODUCTION

The left atrial appendage (LAA) occlusion technique is safe for the prevention of ischemic events in patients with non‐valvular atrial fibrillation (AF), and is an increasingly common procedure.^1^, ^2^ Efficacy and safety has been demonstrated in randomized clinical trials^3^ and several observational “real‐world” studies.^4^, ^5^ While transesophageal echocardiography (TEE) under general anesthesia remains the most frequently used approach for ultrasound guided implantation,^6^ recent papers have reported that intracardiac echocardiography (ICE) can be used to guide LAAO under local anesthesia. The use of ICE from the right atrial (RA) position has been previously described,^7^, ^8^, ^9^, ^10^ but the visualization of the LAA has been inconsistent and suboptimal because of the right‐sided position of the probe, especially when the atria are dilated. The left atrial position of the ICE to guide LAAO was recently found to be a suitable approach, and has thus been compared with TEE. The results suggest that this new approach is effective and safe, and does not increase procedure‐related complications.^11^, ^12^ However, the authors suggest that there is a learning curve, which indicates the need for a real long‐term development policy for this technique. Moreover, these studies evaluated the feasibility and efficacy of ICE‐guided LAAO closure using a single device (either the Amplatzer Cardiac Plug/Amulet device or the Watchman device) and with a short follow‐up period. Thus, we sought to compare the clinical efficacy and safety LAAO procedures guided by either ICE or TEE, using both available devices, with in‐hospital and mid‐term follow‐up.

## 2 PATIENTS AND METHODS

The participants in this non‐randomized study were prospectively recruited between January 2014 and April 2019. Initially, all patients underwent LAAO with TEE guidance (TEE cohort). In March 2015, after 10 procedures had been performed with a dual imaging modality (ICE and TEE), the ICE technique was used alone for all subsequent LAAO (ICE cohort) to increase patient comfort and facilitate procedural logistics. The dual technique imaging procedure patients were integrated into the TEE cohort. Patients eligible for LAAO were known to have non‐valvular AF (chronic, persistent, or paroxysmal), an increased risk of stroke (elevated CHA_2_DS_2_‐VASc score)^13^ with a history of or predisposition for bleeding (elevated HAS‐BLED score),^14^ along with an absolute or relative contraindication for OAC. We excluded patients with the following criteria: presence of an intracardiac thrombus on the preprocedural TEE or CT (*n* = 4), history of previous implanted device for atrial septal defect or patent foramen ovale (*n* = 2). This study complied with the Declaration of Helsinki and was approved by the ethics committee of the University Hospital of Dijon. All patients gave written consent before participation.

### 2.1 Procedure and transseptal puncture

The LAAO technique using the Watchman or ACP devices did not differ between ICE and TEE guidance, except for the fact that all TEE‐guided interventions were performed on intubated patients under general anesthesia compared with only local anesthesia for ICE‐guided LAAO.

### 2.2 ICE and ICE‐guided LAAO

**FIGURE 1:**
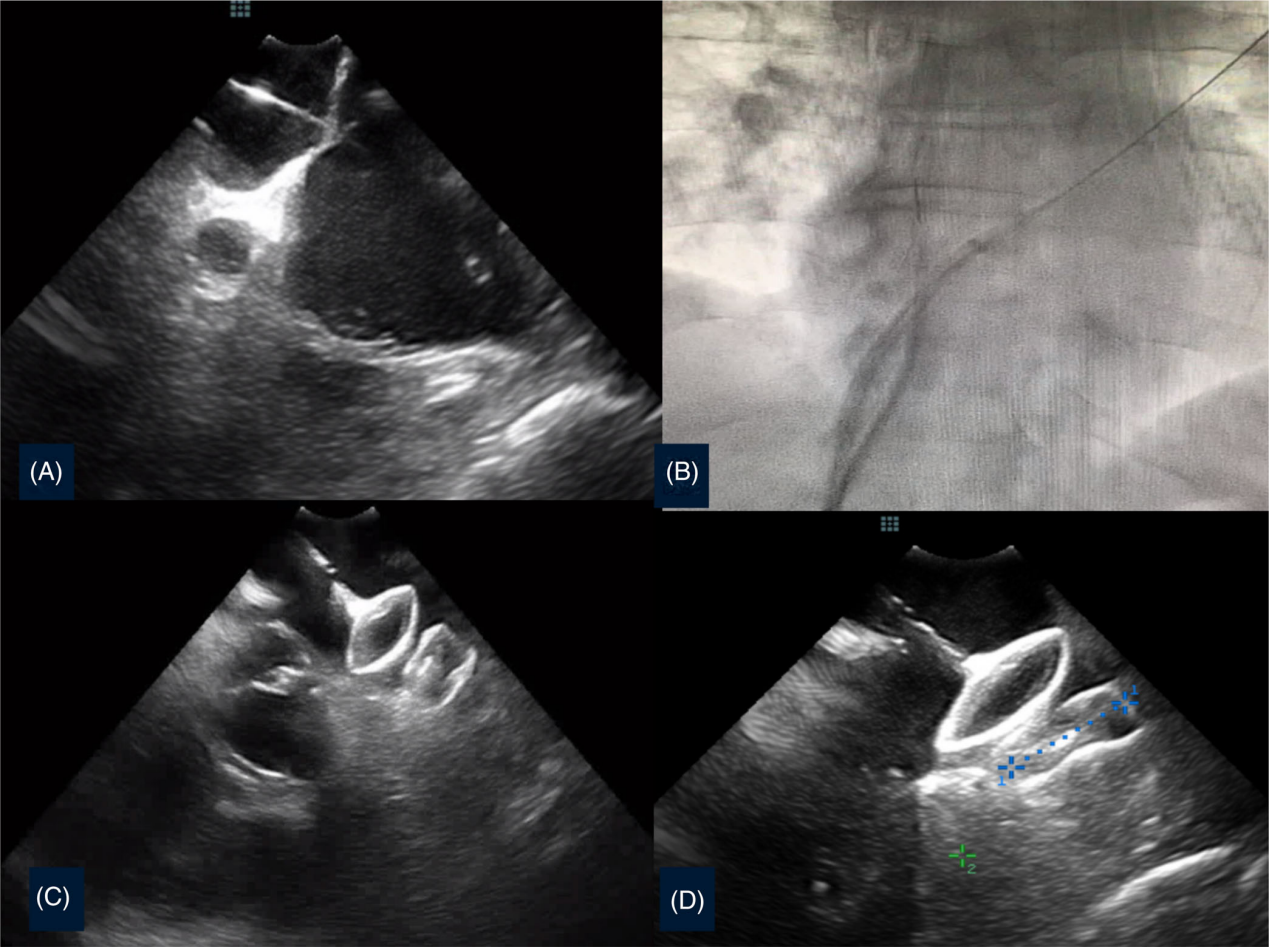
ICE guidance for LAAO. (A) Transeptal puncture in front of the left appendage (ICE probe in the right atrium); (B) angiographic view of the delivery sheath progression after positioning the ICE probe in the left atrium by sliding the stiff wire; (C) stability device position assessment maneuver; (D) check for the diameter of the landing zone part of an Amulet device

All patients, regardless of TEE or ICE guidance, underwent cardiac CT before LAAO for anatomic analysis, device sizing, and exclusion of LAA thrombus. ICE imaging was performed using the 9‐F ViewFlex Xtra ICE catheter (St. Jude Medical) with the Zonare ViewMate Ultrasound Console (St. Jude Medical). All procedures were performed by two experienced physicians (LL, PB) as follows (Figure 1). (1) Single transseptal puncture using the modified Brockenbrough technique under TEE or ICE control; one 8.0F sheath (SL1; St. Jude Medical, Minneapolis, MN) was positioned within the LA. The ICE catheter was positioned in the mid‐RA with a slight posterior flex and a clockwise rotation to obtain the septal view for guiding the transseptal puncture in the inferoposterior part of the fossa ovalis. The transseptal sheath was introduced into the LA, followed by removal of the dilator. A stiff guidewire was inserted through the transseptal sheath into the left upper pulmonary vein. Thereafter, a single heparin bolus (50 to100 UI/kg body weight) was administered to obtain an activated clotting time above 250. The transseptal sheath was exchanged for the device delivery sheath (12 or 14F). (2) The delivery sheath was advanced over the stiff wire into the LA and retracted into the inferior vena cava to dilate the transseptal hole. Thereafter, the ICE probe was aligned using two orthogonal views (Lao and Rao 30°) with the wire, and advanced along the wire into the LA through the same atrial septal puncture hole. A probe position at the entrance of the left upper pulmonary vein was primarily used for device deployment. (3) After placing the ICE probe in the LA, the device delivery sheath was advanced again over the wire into the LA. LAA dimensions were determined using the CT‐based perimeter‐derived mean diameter, compared with selective LAA angiograms in standard angulations (RAO 30/25 caudal, RAO 30/15 cranial) and TEE measurements (45°, 90°). Cardiac CT was performed systematically in both group in order to obtain a reliable assessment of the size of the device. Cardiac ultrasound was used to guide the procedure. The appropriate device size was chosen according to the manufacturer's recommendations. (4) Device deployment under ICE or TEE was performed according the manufacturer's instructions using fluoroscopy. Color Doppler with ICE/TEE and final contrast injection confirmed deployment position and the absence of peri‐device leaks in all LAA lobes sealed by the device. A single femoral vein access was used. A medial 9‐Fr 35‐cm Terumo sheath (Terumo Europe, Leuven, Belgium) was used to introduce the ICE catheter, the transseptal sheath, and later the delivery sheath. The procedure was finalized by manual compression of the femoral access sites. TEE guidance was performed under general anesthesia using the X7‐2t probe with the Philips iE33 console (Philips Healthcare, Eindhoven, Netherlands). The probe was introduced once the patient was anesthetized and intubated, at which time a single venous puncture was performed. Hemostasis was secured for both groups by manual compression at the end of the procedure and heparin neutralization.

### 2.3 Data collection

We collected data relative to comorbidities, including type of AF, prior congestive heart failure, history of CAD, CABG, or TAVR, cardiovascular risk factors such as history of hypertension or treated hypertension, and diabetes. The CHA_2_DS_2_‐VASC and the HAS‐BLED risk scores were calculated with the standard admission variables^13^, ^14^ to estimate individual annual risk of bleeding or stroke. Blood samples drawn at admission were used to assess plasma N‐terminal pro B‐type natriuretic peptide (NT‐proBNP) and creatinine levels. Creatinine clearance was calculated with the Cockcroft formula. Outcomes gathered during and after the procedure followed the Munich consensus paper.^15^ Procedural success was defined according the Munich consensus, which specifies that the device should be implanted in the correct position without device‐related complications and no peri‐device leaks >5 mm on color Doppler. Following implantation, a dual antiplatelet regimen (clopidogrel 75 mg and aspirin 75 mg daily) was prescribed for a 4‐week period, and aspirin was maintained at the same dose. The following safety outcomes were gathered during the in‐hospital period: major periprocedural complications including stroke (hemorrhagic and ischemic) or transient ischemic attack, pericardial effusion requiring drainage, device embolization, major bleeding or access‐related complications. The following clinical endpoints were recorded at a median of 326 (145–869) days after LAA exclusion in our cohort: death, stroke (hemorrhagic and ischemic) or transient ischemic attack, major bleeding, late device embolization and heart failure symptoms during the follow‐up visit. Finally, the rate and degree of residual peri‐device leaks on TEE or CT‐scan performed between 6 and 8 weeks after LAAO procedure were categorized as follows: no leak, small (< 5 mm) or large (>5 mm). No patient was lost to follow‐up.

### 2.4 Statistical analysis

All analyses were performed using Sigmastat Software (Jandel Inc.). The results are expressed as means ± SD or median values (25‐75th percentile) for continuous variables, or as percentages for qualitative variables. The normality of distribution for each variable was analyzed using the Kolmogorov–Smirnov test. Comparisons between the two groups were performed either with an unpaired Student's *t* test or non‐parametric Mann–Whitney U‐test, as appropriate. Continuous variables were expressed as means ± SD, or medians with interquartile range (IQR), and were compared using the Student's *t* test or Mann–Whitney U‐test, as appropriate. Categorical variables were reported as absolute numbers and proportions, and compared using the Chi‐square or Fisher exact test. A 2‐tailed *p*‐value <0.05 was considered statistically significant.

## 3 RESULTS

### 3.1 Procedural features and efficacy

A total of 224 consecutive patients undergoing LAAO were included in the study (175 in the ICE group and 49 patients in the TEE group). Mean age was 76 years, and both groups were predominantly male. The primary indication for LAAO was prior major bleeding events in 91% and 84% of the ICE and TEE groups, respectively. Baseline characteristics were comparable between groups (Table 1).

**TABLE 1:** Patient characteristics

	ICE group *N* = 175	TEE group *N* = 49	*p*
Age, years	76 ± 8	75 ± 7	0.622
Sex (male)	122 (70)	35 (73)	0.990
Hypertension	160 (91)	46 (96)	0.504
Diabetes	60 (34)	10 (21)	0.092
CHA_2_DS_2_VASc score	4.2 ± 1.38	4.5 ± 1.49	0.452
HAS BLED score	4.07 ± 0.99	3.93 ± 1.02	0.184
Paroxysmal AF	51 (29)	11 (23)	0.440
Permanent AF	122 (70)	37 (77)	0.558
Prior **s**troke	122 (70)	31 (64)	0.709
Congestive heart failure	30 (17)	8 (17)	0.633
History of CAD	51 (29)	14 (28)	0.999
History of CABG	10 (6)	5 (10)	0.516
History of vascular disease	37 (21)	11 (23)	0.836
Prior TAVR	3 (2)	4 (8)	0.076
LVEF, %	57 ± 7	57 ± 7	0.942
Creatinine clearance, m**l**/min	66 ± 23	61 ± 22	0.659
NT‐proBNP pre, pg/m**l**	3079 ± 11 567	1993 ± 2973	0.340
NT‐proBNP post, pg/m**l**	2491 ± 9006	2616 ± 3644	0.792
Hs‐**t**roponin post, pg/m**l**	0.29 ± 0.33	0.40 ± 0.33	0.307
Indication for LAAO			
Previous bleeding under OAC	161 (91)	41 (84)	0.778
Cerebral amyloid angiopathy	9 (5)	3 (6)	
High risk of bleeding	20 (11)	3 (6)	
Stroke despite OAC	5 (3)	1 (2)	

*Note*: Data are presented as n (%), Mean ± SD.

Abbreviations: AF, atrial fibrillation; CABG, coronary artery bypass graft; CAD, coronary artery disease; ICE, Intracardiac echocardiography; LVEF, left ventricular ejection fraction; OAC, oral anticoagulant; TAVR, trans‐aortic valve replacement; TEE: trans‐esophageal echocardiography.

Procedural success rates with the device delivered to the appropriate position during the initial procedure was high in both groups, 97% for ICE and 100% for TEE (*p* = 0.895) (Table 2). There was no difference according the device used.

**TABLE 2:** Procedure and post‐procedure characteristics

	ICE group *N* = 175	TEE group *N* = 49	*p*
Procedure			
Procedural success	171 (97)	49 (100)	0.895
Procedural SAE	2 (2)	2 (4.1)	0.590
Total time in the lab, min	66 ± 21	110 ± 27	0.004
Fluoroscopy time, min	21 ± 11	24 ± 12	0.803
Contrast used (ml)	48 ± 42	70 ± 28	0.016
Mean number of devices used	1.15 ± 0.49	1.20 ± 0.46	0.556
ACP device	154 (88)	46 (94)	0.394
16 mm	5	2	
18 mm	4	3	
20 mm	14	5	
22 mm	33	14	
24 mm	25	13	
26 mm	25	4	
28 mm	31	4	
31 mm	11	1	
34 mm	6	0	
Watchman device	21 (12)	3 (6)	0.398
21 mm	4	0	
24 mm	3	0	
27 mm	9	3	
30 mm	4	0	
33 mm	1	0	
**In‐Hospital MACE**			
SAE prior to discharge	9 (5)	5 (10)	0.689
Vascular complication	1 (1)	2(4)	0.755
Cardiac tamponnade	2 (2)	2 (4)	0.532
Device migration	0 (0)	0 (0)	1.00
Device thrombus	3 (2)	1 (2)	0.610
Stroke/TIA	1 (0.5)	0 (0)	0.597
Death	0	0	1.00
**Post‐procedure CT or TEE**			
Device thrombus	1 (0.5)	1 (2)	0.654
No peri‐device leak	134 (77)	39 (80)	0.349
Small leak (<5 mm)	29 (16)	4 (8)	0.306
Large leak (>5 mm)	9 (5)	3 (6)	0.999

*Note*: Data are presented as n (%),or Mean ± SD. SAE: Death, stroke, embolism, major bleed, device migration, major vascular complication.

Cardiac CT interpretation, which was used to select the best device for to the anatomy and diameter of the LA, improved over time. This explains why the first device selected was implanted in 88% of TEE‐guided procedures and 96% of ICE‐guided procedures (*p* = 0.566). Though the fluoroscopy time was similar between groups, the overall time spent in the catheter laboratory was significantly reduced with ICE guidance (*p* < 0.001), facilitating the execution of more LAAO procedures. Finally, the quantity of iodinated contrast media used was significantly decreased with ICE (*p* = 0.028). The length of hospital stay was similar between groups, except for the fact that patients from the TEE group spent time in the post‐anesthesia care unit before returning to the ward, whereas the ICE group returned directly to the ward. The complete exclusion of the left appendage, evaluated either by CT or FU TEE was also comparable between groups during post‐procedure evaluation.

### 3.2 Procedural adverse outcomes

Procedural serious adverse events (SAE) are described in Table 2. In the TEE group, cardiac tamponade occurred in two patients: the first was percutaneously drained, and the second occurred before the device was implanted and required emergency surgery during which the left appendage was surgically closed. In the ICE group, cardiac tamponade occurred in one patient, and aortic puncture occurred in another patient during the transeptal step, leading to surgical closure of the left appendage. Another patient experienced device migration with embolization at the end of the procedure, requiring successful percutaneous retrieval and immediately followed by re‐implantation. In‐hospital SAE occurred in the same proportion between the two groups (Table 2). Interestingly, we observed the same proportion of significant hematoma at access the site but without surgical repair. Pericardial effusion with tamponade occurred equally in the two groups (*p* = 0.532).

### 3.3 Follow‐up adverse outcomes

During the clinical follow‐up, rates of stroke/TIA, major bleeding or device migration were similar between groups. In addition, we found no significant difference in mortality at 30 days of follow‐up in both groups (1.5% vs 1.8%). Finally, we found a trend toward a higher rate of heart failure symptoms in the TEE group compared to the ICE group (16.7% vs. 6%, *p* = 0.053).

## 4 DISCUSSION

In most cases and centers, the evaluation of the LAAO procedures have been done using TEE imaging. The key to successful and safe procedures is an understanding of the transseptal puncture, LAAO size selection for the device‐specific landing zone, and post‐deployment evaluation for leaks and complications. While some operators have developed expertise in using intracardiac echocardiographic imaging for LAAO, this imaging method remains underused. Given the limited randomized data on the topic, we describe here one of the largest studies in the field with 175 patients. There was a recently published meta‐analysis including 391 ICE cases.^16^ In five eligible studies including 1157 patients, the authors found no significant difference between ICE versus TEE in terms of acute procedural success, fluoroscopy time and total procedure time, or complications including pericardial tamponade, device embolization, and stroke. Our results are slightly more favorable to the ICE technique, but our results show overall that ICE was at least as effective as TEE during percutaneous LAAO. Our study also highlights the fact that ICE shortens the time needed for the procedure and requires less contrast agent.

Going forward, the challenge is undoubtedly the learning curve of the LAAO technique under ICE. The use of this approach will require teams to develop a real long‐term policy in order to successfully ensure that training is completed for all the key stages, and especially the transseptal puncture, which remains the riskiest part of the procedure.

Our experience with ICE imaging from the left atrial position using a single transseptal puncture offered excellent guidance for the length of the left appendage, and we were easily able to discriminate thrombus versus sludge in the appendage, and obtain reliable assessment of peri‐device leaks and complications such as thrombus occurrence. Only few data are available regarding left atrium navigation of the ICE probe. The largest study^11^ provided safety and short‐term data on 109 patients who were implanted using an ICE probe in the left atrium with a single transseptal hole. Interestingly, the rate of procedural success was very similar to the rate in our cohort, where either the ACP or the Watchman device was used based on the anatomy of the appendage determined by preprocedural CT analysis, and the demographic characteristics were also very similar. Subsequently, we showed that it is feasible to cross through the same transseptal puncture site with the ICE catheter into the LA while keeping the ICE probe inside the same sheath. Balloon dilatation of the septum was needed for two patients whose interatrial wall was particularly thick. The catheter is first positioned close to the left upper pulmonary vein, providing a long‐axis view of the LAA that is very useful for the placement of the device and measurement of the compression. Maneuvering the catheter toward the right side of the LA and slightly inferiorly allows for oblique imaging of the LAA, which is again very useful for identifying peri‐device leaks. Placement of the ICE catheter directly into the LA to obtain LAA images most closely correlates to both computed tomography (CT) and TEE sizing of the LAA.^8^, ^11^ Though one patient needed a second femoral puncture in the ICE group, we demonstrate here the safety of this procedure when it is performed by trained operators. Moreover, we improved out approach over time by favoring the use of the same sheath to move the two systems, all while using a vascular closure system in order to improve patient recovery.

Finally, LAAO procedures under ICE imaging guidance have the additional advantages of avoiding the risks of general anesthesia or sedation, and of increasing the potential number of procedures performed in the lab. Although an ICE catheter cannot be re‐sterilized, this procedure can reduce direct and indirect expenses related to medications and staff costs.^17^ Because no additional echocardiographer or anesthesiologist is required for ICE‐guided LAAO, hospitals have more flexibility in scheduling and organizing the cath‐lab program. Regarding the increasing number of structural heart procedures expected in the in the coming years, access to general anesthesia should be preferred for procedures in which 3D guidance and respiratory volume monitoring are required.

### 4.1 Study limitation

Our study has several limitations. First, we described a single center experience, so the efficacy of our approach needs to be proven in larger and multicenter cohorts. Furthermore, we excluded patients with a history of atrial septal defect or PFO closure because the manipulation of catheters through the implanted device could be dangerous and induce migration. Imaging guidance was not randomly chosen, but the cohort consisted of two consecutive groups. When learning a new procedure, performance tends to improve with experience, which explains why the results are slightly different for the first procedures with any approach. As the first group underwent TEE guidance, a learning curve may be in favor of the ICE group. On the other hand, a learning curve associated with introduction and handling of the ICE probe is also expected. Nevertheless, from the introduction of ICE approach, it was used almost exclusively in our center. Consequently, our two groups have unequal numbers with potential biases linked in particular to the small number of the TEE group. Because the device sizing was based on preprocedural CT analysis, TEE and/or intraprocedural angiography and not on ICE imaging, no comparisons between ICE and TEE could be made for LAA measurements. Finally, the results are currently limited to procedural, in‐hospital outcomes and one‐year follow‐up, but long‐term follow‐up is ongoing.

## 5 CONCLUSION

Our study provides additional data suggesting that ICE guidance for LAAO, whatever the device used, is feasible and comparable to TEE guidance. Furthermore, ICE guidance precludes the need for general anesthesia or sedation and has the potential to become a preferred imaging modality for LAAO. Left atrial navigation of the ICE probe may also potentially be used to assist other technique such as transcatheter mitral valve replacement.

## CONFLICT OF INTEREST

The authors declare no potential conflict of interest.

## Data Availability

The data that support the findings of this study are available on request from the corresponding author. The data are not publicly available due to privacy or ethical restrictions.
